# Mediastinal Rosai-Dorfman Disease with *KRAS* mutation case report and literature review

**DOI:** 10.1186/s13019-024-02668-0

**Published:** 2024-04-01

**Authors:** Wenyu Zhang, Linan Fang, Jing Wang, Xiaobo Ma, Xintong Hu, Wei Liu

**Affiliations:** 1 Department of Thoracic Surgery, the First Hospital of Jilin University, Changchun, Jilin China; 2 Department of Radiology, the First Hospital of Jilin University, Changchun, Jilin China; 3 Department of Pathology, the First Hospital of Jilin University, Changchun, Jilin China; 4 Genetic Diagnosis Center, the First Hospital of Jilin University, Changchun, Jilin China

**Keywords:** Rosai–Dorfman disease, Sinus histiocytosis with massive lymphadenopathy, Mediastinal diseases, Immunohistochemistry, *KRAS* mutation

## Abstract

**Background:**

Rosai-Dorfman Disease (RDD) is a rare self-limiting histiocytosis, more prevalent in children and young adults. It typically manifests as painless bilateral massive cervical lymphadenopathy but may also extend to extra-nodal sites, with intrathoracic RDD noted in 2% of cases. Distinguishing mediastinal RDD from thymoma on imaging poses challenges, underscoring the reliance on pathological features and immunohistochemical staining for diagnosis.

**Case presentation:**

Patient, male, 33 years old, underwent lung a CT revealing an enlarged round soft tissue shadow in the anterior superior mediastinum, compared to a year ago. Surgical resection removed the entire mass, thymus, and part of the pericardium, confirming RDD on pathology. Genetic testing using second-generation testing technology identified a *KRAS* gene point mutation.

**Conclusions:**

No established treatment protocol currently exists for this disease. However, as genetic mutation research progresses, a novel therapeutic avenue is emerging: targeted therapy integrated with surgical interventions.

## Background

Rosai-Dorfman Disease (RDD), also recognized as sinus histiocytosis with massive lymphadenopathy, is a rare and self-limiting histiocytosis initially documented by Juan Rosai and Ronald Dorfman in 1969 [1]. Predominantly affecting children and young adults, RDD typically manifests as bilateral, massive, painless cervical lymphadenopathy. However, it can also extend to extra-nodal sites such as the skin, soft tissues, bones, upper respiratory tract, and ocular appendages, with only 2% of patients exhibiting intrathoracic RDD [2]. Notably, genetic test results for patients with mediastinal RDD remain unreported. Consequently, we present a unique case of mediastinal RDD and provide a comprehensive review of the clinical, pathological and genetic mutational characteristics associated with this disease.

## Case presentation

**Fig. 1:**
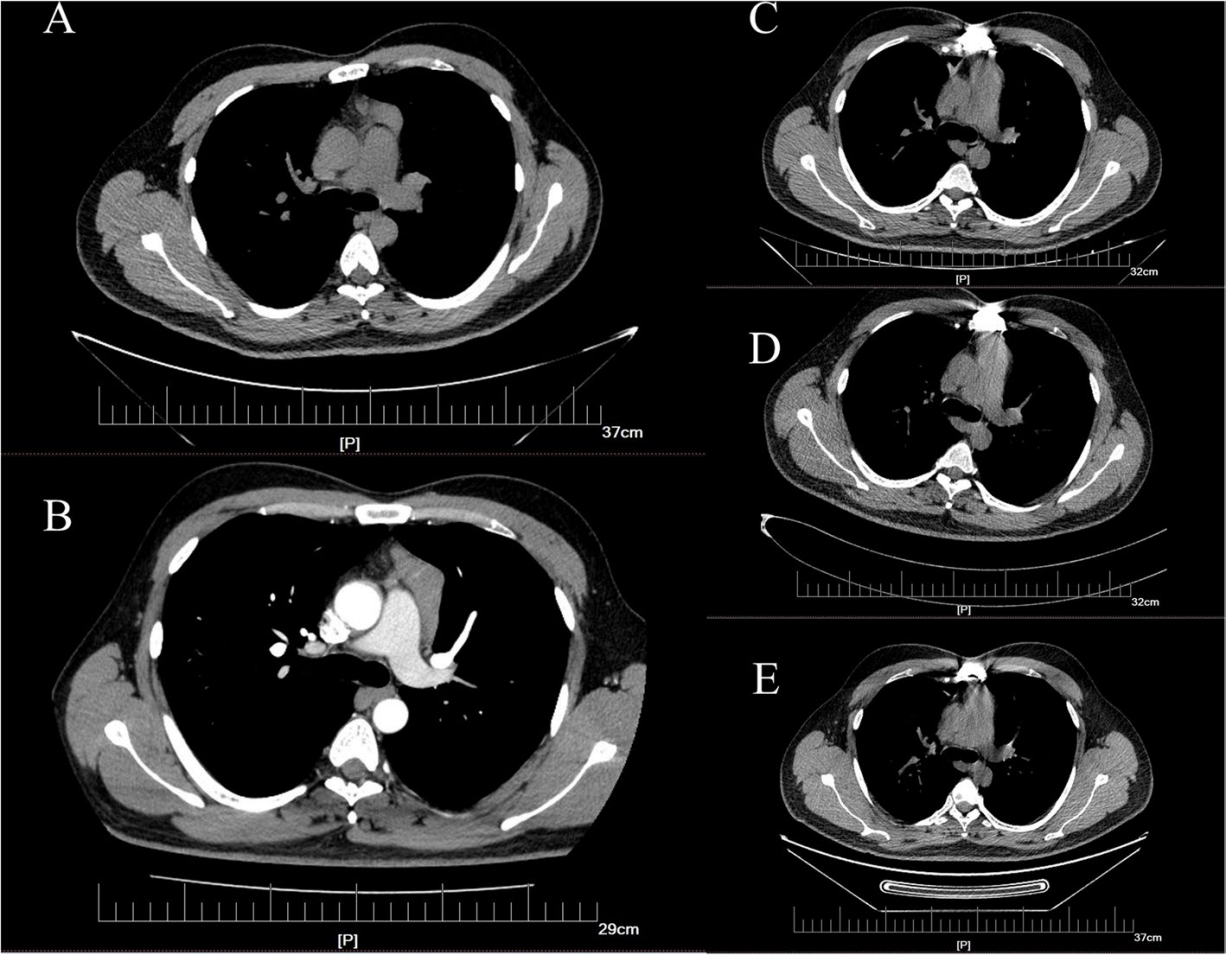
**A** The patient’s chest CT one year before admission, the size of the lesion was about 2.1*2.7 cm; **B** The patient’s arterial stage of chest enhancement CT on admission, with a lesion size approximately 5.3*2.2 cm; **C** The patient’s 3-month postoperative chest CT; **D** The patient’s 9-month postoperative chest CT; **E** The patient’s 2-year postoperative chest CT

Patient, male, 33 years old, admitted with a mediastinal mass identified during lung computed tomography (CT). Notably, the patient had previously undergone the excision of a right retroperitoneal mass in our hospital a year ago, with postoperative pathology suggesting paraganglioma. The patient’s medical history was otherwise unremarkable. One year before admission, the patient’s lung CT revealed a round-like soft tissue density shadow in the anterior superior mediastinal thymus area, characterized by clear margins and uniform density. At that time, it had a maximum diameter of 2.1*2.7 cm and a CT value of approximately 41 Hounsfield units (HU). The mass was observed to be attached to the pericardium, with limited pericardial thickening evident (Fig. 1A). However, a subsequent lung CT post-admission revealed a substantial enlargement of the mass, with a maximum diameter measuring 5.3*2.2 cm (Fig. 1B). The patient reported weakness but denied other symptoms, including fever, night sweats, and weight loss throughout the disease course. Initially diagnosed as thymoma, surgical intervention was performed to remove the entire mass, thymus, and a portion of the pericardium. Subsequent pathological examination of the excised tissue was conducted.

**Fig. 2:**
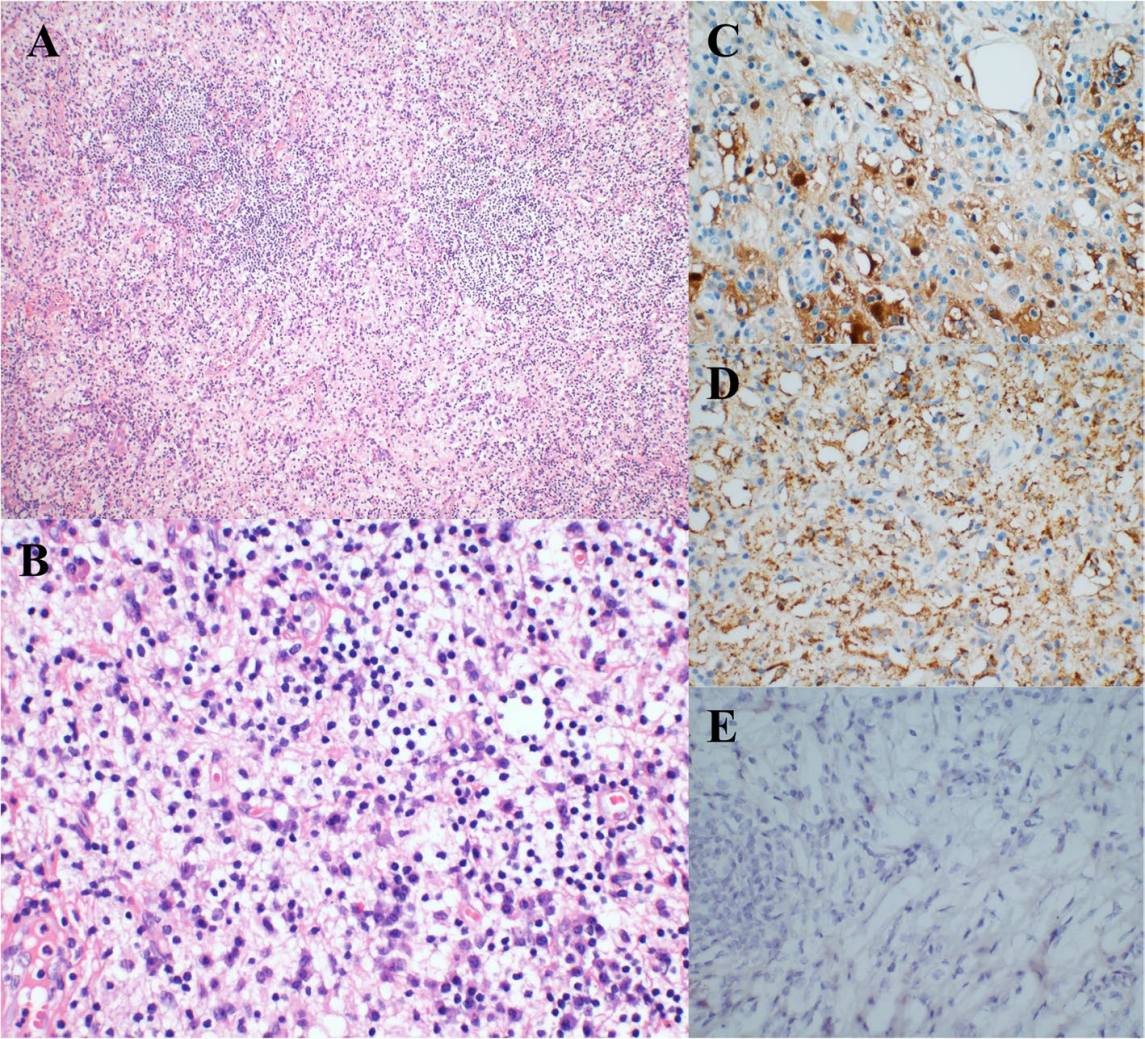
**A** Alternating bright and dark bands were seen under low magnification (HE 100x).; **B** High magnification reveals cytoplasm-rich histiocytes with more lymphocytes and plasma cells infiltrating the periphery; the emperipolesis is visible (HE 400x); **C** Histocyte S-100 positive with visible emperipolesis (IHC 400x); **D** CD68 positive (IHC 400x); **E** CD1a negative (IHC 400x)

Hematoxylin and eosin (H&E) staining at low magnification exhibited an alternating distribution of bright and dark bands (Fig. 2A). Upon high magnification, cytoplasm-rich histiocytes with a significant infiltration of lymphocytes and plasma cells at the periphery were evident, along with the characteristic feature of emperipolesis, indicative of RDD (Fig. 2B). Immunohistochemical analysis revealed positive expression of S-100 and CD68 in the tissue cells, while CD1a was conspicuously absent (Fig. 2C, D, E). The ultimate pathological diagnosis established RDD. Patients also underwent comprehensive genetic testing, encompassing “tumor targeted drugs + chemotherapy drugs + immunogenetic testing 1021,” utilizing second-generation detection technology. This encompassed the assessment of four mutations within 1,021 genes associated with tumor development, including point mutations, small indels, copy number variations, and known fusion genes. The findings unveiled a point mutation in *KRAS*, specifically c.437C > T (p.A146V).

Following a 5-day observation period, the patient was discharged without encountering any complications. Subsequent to discharge, we maintained a scheduled follow-up regimen. Over the course of this follow-up, the patient underwent three repeat lung CT examinations, and the results revealed no discernible evidence of recurrence (Fig. 1C, D, E).

## Discussion and literature review

RDD is a rare histiocytosis, previously categorized as a non-Langerhans cell histiocytosis. However, in the latest classification by the Histoplasmosis Society, cutaneous RDD is now designated within the histiocytosis group C, while other forms of RDD are categorized under the R group of histiocytosis [3].

The prevalence of RDD is notably low, estimated at 1:200,000. Approximately 43% of patients manifest extra-nodal lesions, and intrathoracic involvement is observed in merely about 2% of patients [2]. Specifically, mediastinal RDD accounts for a strikingly minimal proportion, constituting only 0.5% or less of all mediastinal space-occupying lesion [4]. Employing the PubMed database and utilizing the search formula “((thoracic) OR (mediastinal)) AND ((Rosai-Dorfman disease) OR (Sinus histiocytosis with massive lymphadenopathy))”, we conducted a comprehensive search without restricting the time frame. This yielded 142 documents, and subsequent scrutiny in accordance with the PRISMA (Preferred Reporting Items for Systematic Evaluation and Meta-Analysis) guidelines revealed a total of 23 case reports [4–26], documenting 23 patients diagnosed with mediastinal RDD (Table 1).

**Table 1:** Summary of findings in mediastinal Rosai–Dorfman Disease patient

No	Year	Author	Age	Sex	Symptom	Involvement site	Size	SUVmax	Initial diagnosis	Immunohistochemistry	Follow-up	Outcome
1	2023	Liu et al	64	male	NA	anterior mediastinum	1.2*1.0	NA	thymoma	S-100 + , CD68 + , CD163 + , OCT2 + , Cyclin D1 + , IgG4 + , CD1a-	1 year	no recurrence
2	2023	Agarwal et al	57	female	mild pressure	anterior mediastinum, sternum	9.1	34.00	NA	S-100 + , CD68 + , OCT2 + , Cyclin D1 + , CD1a-, Langerin-, and FXIIIa-	9 months	recurrence (left thigh subcutaneous nodule)
3	2023	Lv et al	52	male	NA	anterior mediastinum	6.1*4.6	NA	thymoma	S-100 + , CD3 + , AE1/AE3 + , IgG + , IgG4 +	9 months	no recurrence
4	2023	Dronamraju et al	33	female	dyspnea, myalgias, and chills	middle mediastinum, vertebrae, maxilla, and skull base	2.3*2.1	NA	pulmonary embolism	S-100 + , CD68 + , CD1a-, AE1/AE3-, CD45-, CD30-, Desmin-, Myoglobin-	NA	NA
5	2023	Wu et al	18	male	cough, snoring, shortness of breath	mediastinum	0.9*1.0*1.5; 1.0*1.4*2.5	8.54	NA	S-100 + , CD68 + , HLA-DR + , CD30-, CD1a-, Langerin-	5 months	no recurrence
6	2023	Li et al	41	female	dizziness and headache	posterior mediastinum	6.0	NA	angioleiomyosarcoma	S-100 + , CD68 + , CD163 + , Langerin-	6 months	NA
7	2022	Jia et al	23	male	chest pain	anterior mediastinum	8.5*5.0	8.96	lymphoma or thymoma	S-100 + , CD68 + , IgG4 + , Vimentin + , CD1a-	NA	NA
8	2022	Liu et al	48	female	NA	anterior mediastinum	3.0*2.2	NA	NA	S-100 + , CD68 + , CD38 + , IgG4 + , CD30-, CD1a-, CD123-, CK-	2 months	no recurrence
9	2022	Maniar et al	73	male	substernal chest discomfort	mediastinum, hilus, armpits, abdomen, pelvis	> 6.0	NA	NA	S-100 + , CD68 + , cyclinD1 + , OCT-2 +	NA	NA
10	2022	Oramas et al	40	male	cough, chest pain, shortness of breath	anterior mediastinum	4.0	NA	NA	S-100 + , CD68 + , CD1a-	NA	NA
11	2022	Greggianin et al	61	female	NA	middle mediastinum	2.5*4.2*3.0; 1.7*1.5	5.60; 2.60	NA	S-100 + , CD68 + , CD1a-	NA	NA
12	2022	Nakanosono et al	58	female	NA	anterior mediastinum	5.4*3.3*4.3	NA	NA	S-100 + , CD68 + , CD163 +	5 months	no recurrence
13	2021	Liu et al	28	female	cough	anterior mediastinum	7.1*3.8	NA	thymoma	S-100 + , CD68 + , CD163 + , IgG4 + , CD1a-	3 years	no recurrence
14	2021	Shen et al	49	female	NA	anterior mediastinum	1.7*1.5	NA	NA	S-100 + , CK19 + , P63 + , Cyclin D1 + , OCT2 + , CD20 + , CD79a + , SMA + , CD30 + , CD10 + , CD3 +	3 months	no recurrence
15	2021	Liu et al	48	female	dyspnea	middle mediastinum	4.1*4.9*3.8	7.80	NA	NA	8 months	no recurrence
16	2021	Tsujimura et al	70	female	NA	anterior mediastinum	2.7*1.5*1.2	3.50	thymoma	S-100 + , CD68 + , CD163 + , Ki—67 < 1%	2 years	no recurrence
17	2020	Mohammadi et al	40	male	NA	anterior mediastinum, sternum	8.6	NA	RDD	S-100 +	NA	NA
18	2020	Furia et al	70	male	cough, back pain, fever	mediastinum, left lung, liver, bones	NA	35.00	benign inflammatory granuloma	S-100 + , CD68 + , CD1a-, CD20-, CD3-, CD30-	7 months	volume reduction
19	2010	Kaseda et al	66	female	conjunctival congestion, fever, weight loss	mediastinum, hilum	NA	NA	NA	S-100 +	2 years	no recurrence
20	2010	Brito et al	30	female	cough, chest pain, weight loss	anterior mediastinum	NA	NA	thymoma	CD68 +	NA	NA
21	2009	Costa et al	49	female	cough, dyspnea, chest pain	posterior mediastinum	6.0	NA	NA	S-100 + , CD68 + , CD20 + , CD3 + , CD1a-	1 year	no recurrence
22	2009	Prendes et al	42	female	dyspnea, palpitation	mediastinum	NA	NA	NA	S-100 + , CD68 +	1 year	no recurrence
23	2004	Lim et al	43	male	NA	anterior mediastinum	2.5*1.6	NA	thymoma	S-100 + , CD68 + , CD30 + , CD1a-	NA	NA

CT/MRI plain images of RDD-afflicted patients typically reveal well-defined, irregularly shaped lesions characterized by uniform densities/signals and significantly progressive enhancements on multi-contrast enhancement imaging [27]. All included patients underwent lung CT or enhanced lung CT before biopsy, yielding varying imaging descriptions. Importantly, none received a pre-biopsy RDD diagnosis, and clinicians primarily considered differential diagnoses such as lymphoma, benign and malignant thymic tumors, pulmonary artery embolism, or noninfectious inflammatory disease [13]. This underscores the challenge in leveraging lung CT results for definitive diagnoses, emphasizing their role more in disease screening and guiding the surgical approaches. PET-CT may offer improved insights into RDD indications. In a study by Jia et al. [11], a patient with anterior mediastinal RDD exhibited abnormally high FDG uptake (SUVmax 8.96) on PET/CT, surpassing the average metabolic activity of thymic hyperplasia (SUVmax 1.1), thymoma (SUVmax 2.3) and thymic carcinoma (SUVmax 7.0) [28]. Notably, two reports by Agarwal et al. [6] and Furia et al. [21] recorded even higher SUVmax values at 34.0 and 35.0, indicating a potential reference value for PET-CT in RDD diagnosis.

A definitive diagnosis of RDD typically necessitates HE staining and immunohistochemical analysis. Regardless of the location, RDD is pathologically characterized by sinusoidal hyperplasia of large histiocytes, with extra-nodal lesions often exhibiting a more prominent fibrotic component. Furthermore, in late-stage RDD lesions, fibrosis becomes more prevalent, making the identification of residual RDD islands challenging [29]. While emperipolesis is a crucial indicator suggesting RDD disease, it is not entirely specific, as scattered emperipolesis can be observed in other histiocytic disorders such as Erdheim-Chester disease, yellow granuloma and malignant histiocytosis [30–32]. Meanwhile, immunohistochemistry plays a pivotal role in further delineating RDD disease. The immunophenotype of RDD cells typically manifests as positive for S-100 and CD68, with the potential for positivity in CD163 and CD14. Notably, CD1a is usually negative, serving as a point of differentiation from Langerhans cell histiocytosis [2]. Among all the mediastinal RDD cases included in our study, only one case encountered challenges in obtaining a biopsy sample through surgical resection or puncture. However, histiocytes were observed in all completed pathologies, and immunohistochemistry demonstrated positive results for at least one of S-100 or CD68.

The pathogenesis of RDD remains unclear, with some studies suggesting a potential association with viral infections such as herpesvirus, Epstein-Barr virus, cytomegalovirus, and HIV, but a definitive link has yet to be established [33]. More recently, investigations have revealed mutations in *NRAS*, *KRAS*, *MAP2K1*, and *ARAF* in RDD cases [34–38]. Among the mediastinal RDD cases included in our study, one patient’s mediastinal sample underwent whole exome sequencing, identifying a missense variant in the *IRF5* gene [7]. Another patient showed the presence of a *KRAS* gene mutation, as identified by next-generation sequencing [13]. Additionally, Lee et al. reported a RDD case with the same *KRAS* gene mutation (p.A146V), although the lesion’s location in this patient was not explicitly documented [37]. In our study, patients underwent genetic testing utilizing next-generation sequencing technology, revealing mutations in the *KRAS* gene [NM_033360.2: c.437C > T (p.A146V)] at a frequency of 2.4%. The detection technology employed encompassed four mutation types, including point mutations, small indels, copy number variations, and known fusion genes. The average effective sequencing depth was 1714, covering 1021 genes associated with tumorigenesis and development. The testing strategy included the examination of all exon regions of 312 genes, introns, promoters, or fusion breakpoint regions of 38 genes, and partial exons of 709 genes for somatic mutations. Additionally, all exons of 39 genes were scrutinized for germline mutations, along with assessments for tumor mutational burden and microsatellite instability. Remarkably, our case represents the second instance of identifying *KRAS* gene mutations in mediastinal RDD, offering potential new insights into the etiology of this condition.

There are currently no established standard treatment protocols for mediastinal RDD. Given its often self-limiting nature, conservative management is deemed acceptable. However, among our included cases, surgical resection was undertaken in 21 out of 23 patients for symptomatic relief or definitive diagnosis. The beneficial effects of corticosteroid therapy and radiotherapy were also evident, as reported by Furia et al. [21]. A pivotal study by Garces et al. highlighted that activating mutations in *RAS/RAF/MAPK/ERK* or related signaling pathways may play a role in RDD development [36]. This implies that post-operative genomic analysis and targeted therapy administration could potentially improve outcomes for these patients.

## Conclusion

We documented a case of mediastinal RDD with *KRAS* mutations, exhibiting imaging, pathological, and immunohistochemical features consistent with the characteristics observed in extra-nodal lesions of RDD.

## Data Availability

The datasets used and/or analyzed during the current study are available from the corresponding author on reasonable request.

## Abbreviations

RDD: Rosai-Dorfman Disease
PRISMA: Preferred Reporting Items for Systematic Evaluation and Meta-Analysis
SUV: Standardized uptake value
CD: Scluster of differentiation
